# Comparison of the effects of use, protection, improper renovation and removal of asbestos products on the example of typical old office buildings in Poland

**DOI:** 10.1038/s41598-023-37257-z

**Published:** 2023-08-21

**Authors:** Andrzej Obmiński

**Affiliations:** Instytut Techniki Budowlanej (ITB), 00-611 Warsaw, Poland

**Keywords:** Mesothelioma, Environmental impact, Risk factors, Civil engineering

## Abstract

The study focused on old, German building types “LIPSK” and “BERLIN” used in Poland, since the 1960s in Eastern Europe. The different operations on buildings were analysed: protection and maintenance of asbestos products, asbestos removal and inadvertent damage to asbestos as a result of building renovation. Measurements of respirable (countable) asbestos fibres in the air were carried out using the PCOM + PLM method and SEM–EDS. In the case of the accidental destruction of products, initial contamination was ≈7000 f/m^3^. After 16 weeks from the end of the activity and 20 days of extreme ventilation, contamination decreased to about 500 f/m^3^. At the same time, in similar rooms, without extreme ventilation, the pollution was above 4000 f/m^3^. The average increase in pollution in a dozen or so similar buildings, after asbestos removal in places beyond the work zones, ranged from ≈ 1700–2700 f/m^3^ and lasted for one or two years. These buildings, used without ACM destruction or after product impregnation, have maintained low asbestos contamination < 300 f/m^3^ for more than 10–20 years. So, due to the easy release of asbestos that occurs with any ACM removal and the increased risk of occupant exposure, these works are often inappropriate for the buildings in question.

## Introduction

### Background

In the external environment, PM 2.5, PM 10 and PAHs are important components of pollution affecting the risk of health hazards^1–3^. Environmental pollution with heavy metals is accompanied by the development of transport and traffic^4^. Indoor air pollution in buildings is associated with the use of technologies, construction products and equipment that can release harmful gases, and vapours such as formaldehyde, toluene, benzene, xylene and others, which are contained in impregnants, paints, varnishes—generally, finishing materials in construction. Specialised indoor-air laboratories can find other toxic substances such as phenol cresols, naphthalenes, chlorophenols, chloronaphthalenes and others^5^. A separate category of harmful outdoor and indoor air contamination is fibrous dust—asbestos.

### Health impact assessment

The influence of asbestos on asbestos-related illnesses such as cancers of the respiratory system has been proven. One of the factors modifying the carcinogenicity of fibres is the synergism of asbestos and chemical agents such as PAHs or the compounds mentioned above, which can be adsorbed on the surface of asbestos fibres^6^. Asbestos multiplicatively increases the risk of cancer when a person is exposed to tobacco smoke, but its effect is also confirmed independently of this factor^6–8^. One of the sources of this carcinogenic process at the cellular level is the interaction of reactive oxygen and disruption of intercellular signals causing the initiation, promotion or progression of the neoplastic process^8^.

### The potential effects of intermittent airborne asbestos on users of refurbished buildings

Considering the scope of professional exposure of construction workers and maintenance staff (Removing Building Roofing, Removing Flooring Products, Replacing Ceiling Tiles, Repairing Boilers etc.), the people’s exposure was determined in the range of 10,000–280,000 f/m^3^^9^. Average building airborne concentrations of fibre > 5 um long, registered for USA and United Kingdom buildings, were in the range of 50–400 f/m^3^. From this, it was concluded^9^ that asbestos in buildings does not pose a threat to users and its risk is specified as "Low Exposure Risk”. One of the opinions presented here predicts 4–40 and 6–60 premature deaths due to cancer per million persons if children are exposed to asbestos in schools at levels 200–2000 f/m^3^ and 500–5000 f/m^3^^10^. The environmental asbestos contamination in Poland and the dependence of mesothelioma incidence on environmental asbestos contamination has been well confirmed by numerous researchers^11–16^. When comparing the health effects of permanent, elevated exposure to asbestos, it is worth considering the following example. The concentration of airborne asbestos in the atmospheric air in Poland in the years 2004–2010 was about 480 f/m^3^. In the area contaminated with industrial ACM production before 1990, it was 6000–19,000 f/m^3^. In the period 2002–2008, the average concentration of asbestos fibres in the atmospheric air in the contaminated area of an industrial region was about 1500 f/m^3^. It resulted locally in an excess number of deaths from mesothelioma between 1999 and 2013 (407 cases per million). This indicator was 80 times higher than the national average. In 2016, the incidence of mesothelioma in this area was still higher than the national average (60 times)^13^.

Nowadays, the EU plans to tighten the limit of workers' exposure by lowering the PEL to 0.01 F/ccm. It is worth noting that in the described cases of accidental damage to ACM products during use or renovation, asbestos dust with a similar concentration of asbestos fibres, on the level of 2000–3000 f/m^3^, persists for months or years in occupied rooms.

The condition of these rooms is not controlled for airborne asbestos because the rooms do not contain asbestos (after it has been removed) and have been put into use.

Many countries have introduced a list of occupations in which workers were exposed to asbestos in the past^7^ due to the long latency period of the illnesses mentioned above. Databases (such as “EVALUTIL”^17^) are created containing fibre concentration levels associated with specific jobs and situations. The aim of this article was to do the same.

In the past, asbestos-containing materials (ACMs) were in widespread use. The growing demand for asbestos removal work, the demolition of old buildings, and the elimination of asbestos equipment and installations have resulted in an increase in errors and poor workmanship. Examples of high concentrations of asbestos dust in asbestos work zones caused by improper procedures and lack of appropriate technical means are provided in the literature^18, 19^. These works have become more and more popular and routine. They are covered by applicable national programmes. Their quality and control are declining due to economic savings pressures. Simultaneously, some countries lack air pollution limits after asbestos removal work is completed and/or during the building's use, making it difficult to detect defective work.

Most of the national regulations focus on the conditions for carrying out these works with asbestos because its disassembly is associated with the mechanical destruction of these products. This causes high concentrations of asbestos aerosol to be created in the workplace and workers are exposed to high exposure. The literature reports significantly different values of dust concentrations in the tested air. In some industrially developed areas of Iran, high concentrations of up to 26,000 f/m^3^ are recorded outdoors^20^, In the indoor air of buildings with asbestos-containing products, much lower values < 2000 f/m^3^ were recorded in Italy^21^, however, dust concentrations measured in other countries, facilities and other techniques may significantly differ from these data (Tables 1 and 2). It depends on many factors, such as the technical condition, the form of use of the premises, the degree of destruction of products, etc. How are the values measured in Poland compared to this? The presented article shows the values recorded in different buildings, different conditions of exploitation and in the incorrect manner of asbestos removal.

**Table 1:** List of asbestos fibre concentration ranges according to literature data.

Type of measurement		Indoor air measurement site-building		Analysis method	Concentration range of asbestos fibre [f/ m^3^]	Literature information [references]
1	3	Korean town-country school buildings, randomly selected		PCM	3000–6000	(Yoon et al. 2011)^22^
		General construction (including schools, residential buildings, and offices) in normal use		TEM	120–300 respirable. Fibre 10,000 structure^1^/m^3^ [s/m^3^]	(Lee et al. 2007)^23^
		The concentration of asbestos dust released from efficiently operating heating systems, using a soft insulation board, simulating operational disturbances—impacts, vibrations		TEM	** < **50	(Burdett 2016)^24^
2		Buildings with poor condition of asbestos products		TEM	730	(Report SRC, Inc. Denver, CO 2013)^25^ (Darcey 2014)^26^
		Buildings with good condition of asbestos products			590	
		General construction		TEM	40–2000, on average 200–500	(Health Effect Institute (Asbestos Research, Cambridge Upton A. C. 1991)^27^
		Zones with damaged ACM products in buildings		TEM	7,000–8,000 s/m^3^	(Reynolds et al. 1994)^28^
3—4		ACM roofing removal (suitable for asbestos cement boards with 5–10% asbestos) area samples		PCM	“Sample area" 600–16.000; "Samples individual" 9,000–30,000	(Lange, Thomulka 2000)^29^
		Destruction of asbestos and asbestos-cement products (plates); folding, manipulation of disassembled waste material (in dry techniques) in from	- roof	PCM	100,000–300,000	Brown (1997)^30^
			- facade panels		< 100,000	
			- renovation of the cover roof		100,000–200,000	
		Demolition of houses with asbestos-cement products (roofs, facades) within 1 month from the completion of works and measurements carried out during the project		PCM/ SEM	50,000–400,000	Kakoei (2014)^31^
		Measurement with individual dust meters when removing products,		PCM	3,000,000–20,000,000	Dufrense (2009)^32^
		Stationary measurements—in the "area" of working with asbestos			2,900,000	
		Estimated level of asbestos fibers exposure to workers during improperly performed disassembly works of ACM sprayed products using BALF^2^		PCEM	An extrapolation considering the duration of exposure: 100,000,000	Dumortier (2012)^18^

^1^TEM counted structures—fibers and fiber associations below the dimensions and geometries assumed for the respirable. Fiber [s/m^3^].

^2^BALF- mineralogical analysis of Broncho-alveolar lavage fluid.

(1) Normally used buildings containing asbestos (use of good condition, undamaged products without removing, destroying, disturbing or processing them).

(2) Buildings with damaged asbestos products.

(3) Zone with ACM, during disassembly or repairs, the so-called "area" tests.

(4) Individual asbestos fibre measurements, carried out at work stations (directly in the breathing zone).

**Table 2:** Evaluation and recommendation as an effect of work on Occupational Exposure Limit for asbestos, based on scientific reports for exposure limits to asbestos in the workplace.

*Air concentration of asbestos [F/m^3^]	Excess lifetime cancer risk (cases per 100,000 exposed)	ECHA report^33^
1000	1.2	
2000	2.5	

*Assuming exposure of 8 h per day, 5 days per week, over a 40-year working career (starting at 20 years) and calculating risk until 89 years of age.

### Purpose of the study

Currently, the opinion among some asbestos specialists is that ACMs products must definitely be removed instead of preserving them. Others argue the solution to the problem should depend on the characteristics of each individual object. However, wrong techniques and methods of asbestos removal are often caused by economic factors. In some cases, the errors made are difficult to detect and pose a threat to users and bystanders. The aim of the study was to estimate the effects of execution errors when working with asbestos-containing materials or their normal use. Contamination in zones distant (in place and time) from the work zones is shown and compared. These are spaces that are not subject to the normal controls of contamination by asbestos fibres. However, the literature on the subject proves that even a small scope of work can cause a high concentration of asbestos aerosol.^28–30,34,35^

The data provided may therefore constitute a database of information on the exposure of people outside the work zone, in particular in the case of errors made by asbestos contractors. Showing that threat to users and residents was the main purpose of the article. In some countries it is an unconscious threat to those at risk, it is omitted from the applicable regulations and procedures for implementing works and in the final inspection of works.

Such research has its limitations. Tests carried out on the outside air surrounding the building, as well as in rooms with large cubic capacity, are characterized by a large dispersion of results. The same is observed in buildings of different technical conditions and with large ACM damage. This is due to local changes (in a given place and time) in the concentration of asbestos aerosol mixing with the surrounding air. So we are dealing with a mixture of fibres in the air (not a chemical solution). For this reason, the results of such tests performed in the same place at short intervals may vary, and the values obtained should be treated as approximate. In addition, it is difficult to recognize the types of asbestos in air testing. Only chrysotile was identified in the material samples (Supplementary Information 1, d_6_–e_6_). Only chrysotile, the dominant asbestos in ACM products, was recorded in the air samples too.

### Literature review and relation to the data

The values of airborne asbestos fibre concentrations (asbestos risk category) in the literature present different levels in four categories of values according to Table 1 and the type of exposition^6^.

Projected estimation of risk by ECHA (European Chemicals Agency) is presented in Table 2.

The studies that describe a “5” category for asbestos airborne fibres exposition, which should be investigated. It is mostly a variable level of about a few thousand f/m^3^ but it can be much higher. It is the result of errors at work in buildings containing ACM. It applies especially to people who are not directly involved in the work with ACM, but who are within the exposure range or near the work environment. The results of "faulty works" carried out using "dry" or “DIY” techniques were reported by Brown^30^ and others^36,37^. Other improper removal techniques were described by Dumortier^18^. These results give a wide range of exposure levels. In the case of disassembly works with asbestos-cement products, concentrations of 0.1–0.6 f/ml were recorded, while in the case of incorrect removal of asbestos products using “spray on”, up to 100 f/ml were observed. The quoted literature values refer most often to personal measurements. Those are much larger than the results of "area" measurements, obtained in the used rooms, measured after some time has passed from finishing work with asbestos.

Generally, the assumption was that a damaged product (material) with asbestos presented a higher risk of asbestos fibre concentration in the internal air than in the outdoor air^26^. Such concentration was also determined by many factors, including the method of tests and the kind of removal of asbestos and dust from the internal space. The relationship between the magnitude of airborne asbestos fibre concentration and the scale of damage, Refs.^34,35^ is conditioned by many individual building situations and building characteristics independent of ACMs conditions.

The measured concentration of fibres varies depending on the activity of new dust emission sources, the time that has passed between their formation and sampling, and the building’s use, ventilation and general condition^34,35,38^. Research leads to conclusions^30,33^ that exposure to historically high airborne fibre levels, prevailing half a century ago, may still occur today when special work procedures are not strictly applied.

## Materials and methods of analysis

### Tested buildings and their contamination in the author's research

**Figure 1:**
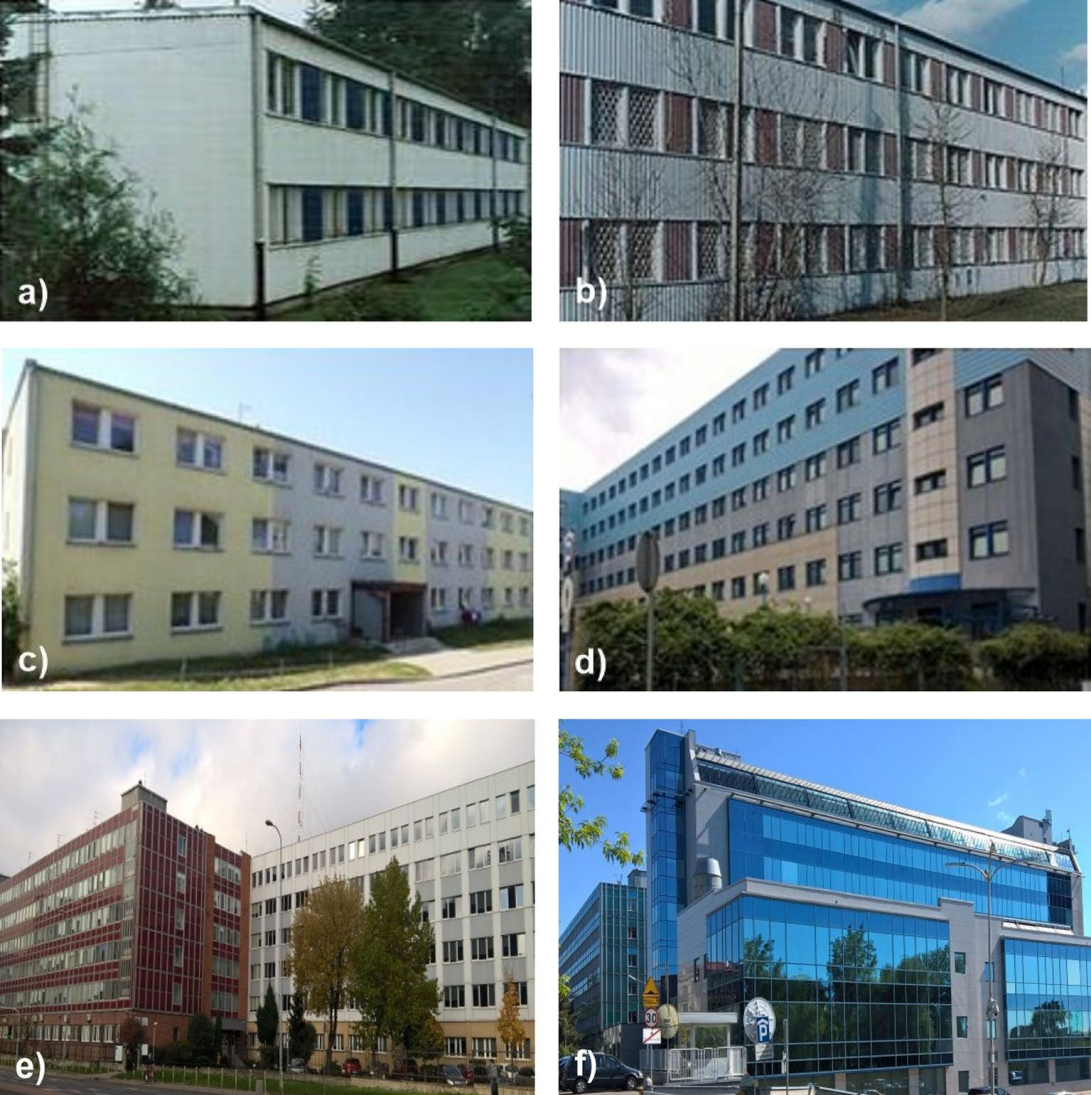
The tested buildings. Figure: (**a**) and (**b**) Typical “BERLIN” building, general view before renovation (Poland, Warsaw), (**c**) Current view of building type “BERLIN” no. 2 after correct renovation (enclosure and encapsulation) (Poland, Olsztyn), Buildings type “LIPSK” in Poland, Warsaw: (**d**) Building type “LIPSK” after similar renovation as building type “BERLIN” no. 2, (**e**) Poland, Warsaw, Complex of two connected buildings of type “LIPSK”, on the left, building no. 11 (red), on the left side, building no. 7 (white) on the right side, (**f**) Poland, Warsaw, Buildings type “LIPSK” after asbestos removal and change of façade. Inside, on the left – “LIPSK” without any modification, removalor replacement of construction products. Typical damages of “SOKALIT” are shown in Supplementary Information 1 and 2.

A typical lightweight construction of popular German buildings was chosen for the study. Types “BERLIN” and “LIPSK” were perfect candidates, as they are quite old and often subjected to renovation or demolition. The tested buildings are shown in Fig. 1.

Building type “BERLIN” (Fig. 1a–c) is a small building, without a basement. It was produced in former East Germany in the middle of the twentieth century. This popular building contained asbestos materials in different places: curtain walls (A1, d_6_), exterior walls and partly in other partitions in the form of friable asbestos board elements. Buildings could differ in configuration and the number of ACMs used. There were fireproof boards, called "SOKALIT", and typical asbestos-cement boards, named “GLAGIT” used partly as non-friable façade cladding. On the inside, the building was finished with “SOKALIT” boards. Asbestos-containing products are in direct contact with the internal air. “SOKALIT” A1, a–d_2_ boards contain approximately 18–20% chrysotile asbestos. The area of asbestos-containing material is ~ 820 m^2^, which corresponds to approximately 15 tons of the product. On the outer wall, from the outside, there is a non-friable asbestos–cement product containing 13% chrysotile asbestos, covered in corrugated sheet metal. Dozens of such buildings have been tested and described as part of ITB’s research^37,38^. Two- or three-story buildings of this type designed for the construction backup have a light, non-rigid (steel) structure, with external walls and internal curtains sandwich walls. This building’s construction and it’s ACM products are similar to its bigger "brother" building, type “LIPSK” (Fig. 1d,e). This type contains about 48 tons of similar ACM products (Supplementary Information 1, d_3_, d_4_).

**Figure 2:**
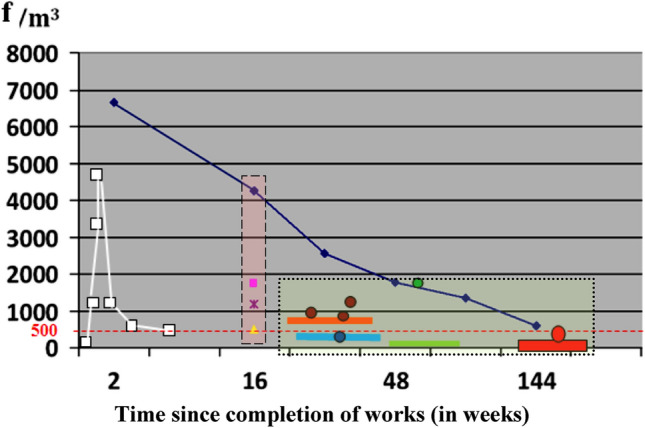
Comparison of airborne asbestos concentration in building types “BERLIN” and MOA – during demolition. The dashed white line corresponds to changes in pollution with the complete demolition of the MOA building. The navy blue line shows changes in indoor air pollution in the “BERLIN” type building after improper renovation, which led to damage to ACM products. The measurement was carried out up to 144 weeks from the moment of suspension of works. Only the last measurement reached the level of pollution that analogous buildings had without asbestos removal. The rectangle with a pink filling shows the values measured in this building 16 weeks after the renovation was discontinued. The rectangle with a green filling, 4 examples of asbestos dust concentration values in buildings of the same type (“BERLIN”), in which asbestos has not been dismantled, are circled. Markings: orange and brown colors correspond to the building before ACM was treated with impregnating agents. Blue and green colors with a lighter and darker shade - buildings after applying the impregnating agent. Red color - building without renovation in good technical condition. In the figure, the horizontal segments represent the average value of the concentration of asbestos dust in the building, the circles - the single values in the rooms exceeding the average value.

**Figure 3:**
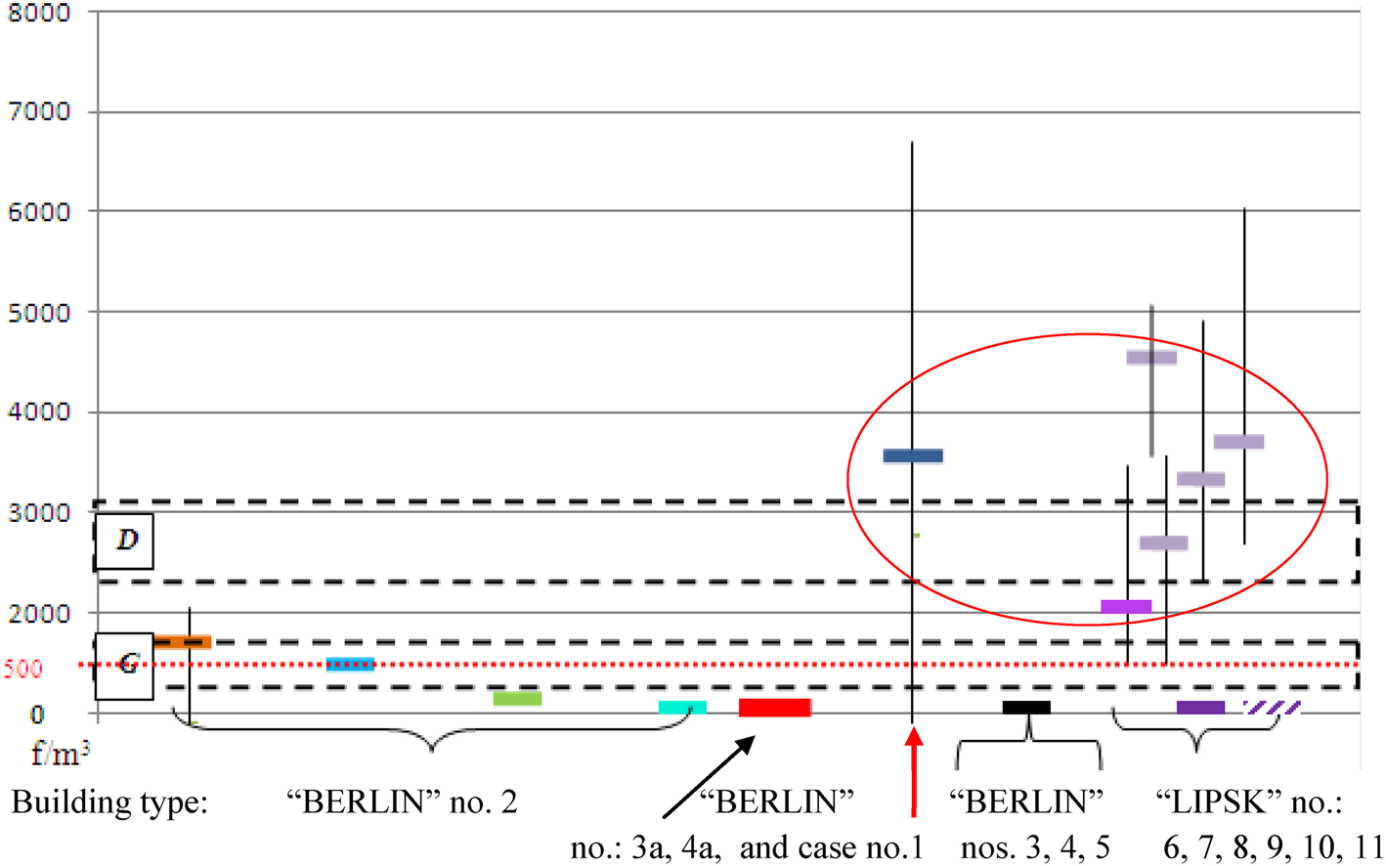
Building no. 1 After 2 weeks of the wrong renovation:  Renovated rooms after prolonged intensive ventilation.  Rooms with damaged ACM-s, not renovated, in use.  Renovated rooms not in use. Renovated rooms, normally used from two weeks to three years after completion of work.  The trend of contamination changes. Building type “MOA” no. 11 During the wrong demolition: Single measured result in the average room.  The trend of changes in pollution. Building “BERLIN” no. 2 A properly renovated and the others, without renovation, nos. 3, 4, 5, 3a and 4a:  Average air analysis results for the entire building in 2002 (before renovation). Use without ACM restrictions. Apartment No 19. Average air analysis results for the entire building in 2006 (1 year after renovation).  Apartment no. 19.  Average air analysis results for the entire building in 2015 (10 years after renovation).  Apartment No 19. Analysis containing the premises in which the owner, after renovation, in 2006 rearranged the internal walls on his own (in Table 3 these are the values of 1300 and 1800 f/m^3^).  Average air analysis results for the entire building in 2022 measured results in average room analyzed in optical microscopy and SEM—EDS method (10 measurements).  Buildings type “BERLIN” no. 3, 4, 5—not renovated, good condition of ACM. Average air analysis.  Buildings type “BERLIN” no. 3a, 4a,—not renovated, different condition of ACM, local minor damage. Average air analysis.  Average pollution in rooms with the greatest usable damage of external walls. Buildings “LIPSK” nos. 6 – 11:  Building type “LIPSK” no. 6, average air analysis results after correct preservation of ACM-s.  Building type “LIPSK” no. 8–10, average air analysis results outside of leaky work zones during asbestos removal in these zones.  Building type “LIPSK” no. 11, average air analysis result in rooms during asbestos removal in building no 7. Both buildings are connected and were separated during renovation.  Building type “LIPSK” no. 7, average air analysis result in rooms, 10 month after finished asbestos removal and final cleaning of all the building.

The author’s results of the tested buildings in the form of concentration values of respirable asbestos fibres are presented in Tables 3, 4, 5, 6 and 7 and in diagrams, Figs. 2 and 3.

**Table 3:** Summary of test results of an uncorrected renovation, Building type “BERLIN” no. 1.

Description of the rooms	Number of tests	Average [f/m^3^]	Max [f/m^3^]	Min [f/m^3^]	Std. Dev σ [f/m^3^]
All rooms, renovation carried out in the past, three years back	6	950	1760	430	500
Not renovated rooms	5	1200	1600	860	300
Rooms with varying significant wall damage	2	5000	5550	4600	670
Renovation completed two weeks before the test, used	5	3200	6700	570	2000
As above, after 20 days of the intensive airing of the rooms (ventilation by opening the windows), 16 weeks after the completion of renovation	3	500	630	440	100
All rooms	21	1880	6700	430	1870

**Table 4:** Building type “BERLIN” no. 2: before correct renovation and after several years.

Tests carried out in the year	Description of the rooms	Number of tests	Average [f/m^3^]	Max [f/m^3^]	Min [f/m^3^]	Std. Dev σ^1^ [f/m^3^]
2022, after 17 years of completed correct renovation	Standard use of premises including rooms in apartment 19	10/OM 4/SEM–EDS	< 300 < 170	< 300 < 170	0 < 170	– –
2015, after nine years of completed correct renovation	Correcting the use of the rooms after renovation	40	< 300	770	0	165
	Rooms in apartment no. 19, with an unprofessional adaptation of interior walls by the owner of the rooms	2	1500	1800	1300	424
	All rooms	42	< 300	1800	0	330
2006, one year after renovation (after 38 years of use)	The renovation carried out during the use of rooms	6	470	730	< 300	190
	Rooms in apartment no. 19	1	340	–	–	–
2002, before any renovation or adaptation, (after 35 years of use)	Asbestos fibre concentration in the rooms before renovation	10	930	2050	< 300	540
	Rooms in apartment no. 19, before the unprofessional adaptation of interior walls by the owner of the rooms	3	1200	1300	1040	150
	Other rooms, except apartment no. 19	7	830	2050	< 300	611

^1^Stand. Reverse σ [f/m^3^]. The analysis includes all the rooms studied (21 flats, including 42 rooms). A separate case where the owner of two rooms, after renovation, rearranged the internal walls, was excluded.

**Table 5:** Other “BERLIN” type buildings, tested in 2002–2010, had good conditions of ACM, walls of the rooms were only painted.

Description of rooms		Number of tests	Average [f/m^3^]	Max [f/m^3^]	Min [f/m^3^]	Std. Dev σ [f/m^3^]
Asbestos fibre concentration in the rooms	Building no. 3	8	< 300	300	0	90
	Building no. 4	11	340	800	0	200
	Building no. 5	9	< 300	500	0	140
	Building no. 3a	30	150	560	0	–
	Building no. 4a	30	70	300	0	–

**Table 6:** LIPSK” type buildings.

Asbestos fibre concentration in the rooms / Description of the rooms	Number of tests PCM + SEM–EDS	Average [f/m^3^]	Max [f/m^3^]	Min [f/m^3^]	Std. Dev σ [f/m^3^]
“LIPSK” no. 6/chosen rooms on all floors	18	< 300	< 300	0	–
"LIPSK" no. 7, rooms on the ground floor (during removal asbestos works not on V floor)	6	3000	5700	1800	1824
“LIPSK” no. 11 during all asbestos removal works in “LIPSK” no. 7	14	1200	2700	320	790
“LIPSK” no. 11 during the asbestos destruction process	8	1500	2700	320	970
“LIPSK” no. 11 after asbestos removal works in “LIPSK” no. 7, after seven days of intensive ventilation by opening the windows	6	800	1000	660	130
“LIPSK” nos. 8–10 measurements outside of leaky work zones during asbestos removal in these zones	45	2800	4500	500	–

**Table 7:** Building no. 12, type “MOA” (similar to type “BERLIN”). Changes indoors and environmental pollution as demolition work progresses.

Days of work	1	2	3	4	7	13	14	15	16	18	19	Place of measurement
Advancement of works	a	b	b	b	c	d	e	f	g	g	g	
Fibre concentration [f/m^3^]	< 300	< 300	< 300	< 300	< 300	400	600	2200	600	300	< 300	10 m from the works outside the building
	300	–	–	300	1200	3600	4800	1200	600	700	600	Inside the demolished “MOA” building
	< 300	< 300	< 300	< 300	< 300	< 300	< 300	< 300	< 300	< 300	< 300	Inside the other building, 30 m away from the works

Progress of work: a—before the renovation, b—preparation zones of work, c—the start of disassembly, d—dismantling of walls, e—removal of the roof, "unsealing" of the building, f—final works, g—waste disposal. On the 19th day, measurements “inside the demolished building" were carried out on the foundations and the remains, i.e. in the atmospheric air not limited by walls.

Building type "BERLIN" was analysed in the following cases. Building no. 1: general renovation works were carried out in this building without planning for asbestos removal and without awareness of the accidental asbestos disturbance. Were ACMs slightly damaged? It seems that these activities were less destructive than removal, while at the same time having a random severity greater than normal operational damage. These included replacing windows, insulating the façade, interior painting and removal of previous paint along with sandblasting the walls, plastering cracks etc. The interior of building “BERLIN" no. 1 and the condition of the ACM after interrupted renovation, which corresponds to the results, are shown in photos (a, b, c_1_, c_2_, d_1_), in Supplementary Information 1 and in photos (a–e) in Supplementary Information 2. The results of tests on this building are presented in Table 3. For comparison, the same type of building, which was properly renovated, building no. 2, is presented Fig. 1c. The renovation was carried out to protect the external ACM boards (asbestos-cement boards) and the internal ACM boards, “SOKALIT”. As part of this renovation, the building was covered with polystyrene boards with a new façade on the outside. The plasterboard had been glued and painted to the internal “SOKALIT” boards. All works were started and completed in 2005. The results of the tests are shown in Table 4.

The same types of buildings nos. 3, 4 and 5 were in good condition and used for several years without significant ACM damage. The research was conducted during their normal operation over several years. The results are presented in Table 5.

The results of buildings nos. 3a and 4a are presented in Table 5. Damage of ACMs in buildings nos. 3a and 4a are similar to those in building no.1. However, the results are different.

Buildings of type "LIPSK" are a good example of leakage and dispersion of asbestos fibres in the air inside a larger building due to various circumstances. A common one is a space above the suspended ceiling and between the layers of the sandwich walls. This allows dust to be easily transported throughout the building. The results of “LIPSK” analyses are presented in Table 6:

- Building no. 6, tested in 2022 after 20 years had passed from the completion of correct preservation of ACM (Fig. 1d). The renovation was carried out in the same way as in "BERLIN" 2.
- Buildings nos. 7, 8, 9 and 10 were tested during the removal of asbestos, outside the work zone, in places 30–100 m away from the works, and inside the building.
- Building no. 7 was tested during asbestos removal, outside the work zone, on the ground floor, about 80 m from the work zone, while the asbestos work was in progress on the 5th floor (Fig. 1e**,** with a new white façade).
- Building no. 11 is connected to building no. 7. There was no ACM destruction work carried out. Tests were conducted during and after asbestos removal in building no. 7 and one week after the intensive ventilation in building no. 11.

The last building, "MOA", type no. 12, was similar to the "BERLIN" type. "MOA" was monitored during complete demolition, without prior removal of ACMs. Changes in indoor and outdoor air pollution resulting from the processes of settlement, displacement and dispersion of airborne asbestos fibres are shown in Table 7. As in the case of building no. 1, the contractors were not aware of the presence of asbestos inside the building (photos showing the degree of ACMs destruction in Supplementary Information 3 and 4).

All studies concerning works were carried out in Poland between 2002 and 2022. The strategy for selecting the test sites and the method of sampling was in accordance with the International Standard ISO 16000-7.

### Microscopic analysis terms and conditions of sampling

Microscopic analyses: PCM + PLM methods (phase-contrast and polarised light microscopy). This method has been previously, repeatedly verified by electron microscopy and comparative inter-laboratory studies. SEM–EDS was used to perform some tests according to ISO standards^40,41^. Those results could be compared to historical tests and exposure and dose–response estimates.

The investigator personally analysed the conditions of the tested buildings and carried out air tests at various stages of building use in terms of the concentration of asbestos-respirable fibres (countable asbestos fibres according to WHO criteria, L > 5 µm, Ø < 3 µm and L:Ø < 3:1).

Sampling was carried out in accordance with the standards and the purpose of the study according to standard ISO^41^ was to determine the concentration of asbestos dust in the used buildings.

The windows were closed during air sampling in the used buildings, though room use during the measurements (movement of employees) caused the mixing of air from different rooms covered by the study. In-office fans were used to standardise the tested air within individual rooms, which were still in use during sampling. This is called "dynamic" sampling, which activates settled dust and increases the possibility of recording all released dust during the test. For the optical microscope, the air samples were collected on filters made of Millipore AA cellulose esters with pore diameters of 0.8 µm. A flow volume of tested air of approximately 1.5 m^3^ was ensured through each filter for 2 hours. After chemically treating the filters, (the filters were made transparent in hot acetone vapours and stabilized in triacetin) microscopic tests were performed, calculating the number of counted respirable fibres (PCM method), identified as respirable asbestos fibres (PLM method) per 1 m^3^ of internal air. During microscopic analysis, the phase contrast technique according to the NIOSH 7400 method was used. The observation of each of the counted respirable fibrewas supplemented with its identification using light polarisation (based on optical features). Magnifications used: 500 × (or 1000 ×  + immersion). The number of observations of a single filter was approximately 4 × greater than the NIOSH standard, which increased the sensitivity of the determinations. The adopted limit of quantification for this method is 300 f/m^3^. The described method is a PCA-accredited research procedure and used at the Instyutut Techniki Budowlanej in Warsaw^42^. The expanded uncertainty of the results, (determined in the computer program developed for the laboratory specifically for these tests), amounts to approximately 20–30%. The applied analysis method has been described previously^34,35^. Two techniques of air sampling were used: static (natural, stagnant conditions, where the ambient air was not additionally mixed—samples were taken from indoors when the ACM products were still in the used rooms) and dynamic, only in unused rooms, where asbestos was not being dismantled, but strongly disturbed. The air was mixed using fans to activate settled asbestos dust. The selected research technique allows for the comparison of the historical results presented here and by other researchers, aimed at the assessment of threats. However, it does not pretend to be as precise as the TEM technique^43, 44^. For SEM microscope analysis, the air samples were collected on filters (gold filter coated with gold on both sides with a layer thickness of 40/20 nm in accordance with VDI 3492/ISO 14,966—diameter 25 mm, pore size 0.8 μm). Instruments used: AS50 aspirators with a flow rate of 0 50 L/min; optical microscope for phase contrast and light polarization Carll Zeiss Jena JENAPOL (SEM) produced by Zeiss, model Sigma 500 VP (Carl Zeiss Microscopy GmbH, Köln, Germany). Secondary electron (SE) and backscattered electron (BSE) images were collected. Phase compositions were analysed using the EDS detector model Oxford Max 40.

### Ethics statement

Experimental animals and human participants were not used in the studies. No human or animal samples or studies were used in this study. All the studies concerned "inanimate nature". These were studies of buildings, building materials, indoor air and air pollution. A statement to confirm that all experimental protocols were approved by a Building Research Institute (ITB) in Warsaw and Polish Center for Accreditation (PCA).

## Results

Only chrysotile fibres were detected in the air samples. Figures 2 and 3 graphically show a comparison of indoor air pollution in “BERLIN” and “LIPSK” at different phases of the operation process. The colours in the common legend were used to designate the buildings. The average test results in all described cases in rooms with poor technical conditions of ACMs and work errors were characterised by a large dispersion of fibre concentration at the Std. Dev. level σ ≈ 50% for the average value of the obtained results. The reason may be different conditions of re-emission of originally settled dust into the air and their persistence in the form of an aerosol, as well as the presence of active sources of dust emission from damaged products.

Figure 2 presents tests of the "BERLIN" building during different cycles of use and “MOA” during demolition. Buildings nos. 1 and. 12, were measured after the destruction of ACM over time, up to 144 weeks from the completion of all works. Additionally, for comparison purposes, changes in contamination of indoor air in "BERLIN" building no. 2 during 20 years of use, and buildings nos. 3a and 3b, were meureasd. Tests for building no. 2 were made before and after ACM impregnation, with the marking of the apartment in which the internal walls were moved (apartment no. 19). The test results for "BERLIN" building no. 2, 3a and 4a present in Fig. 2 are not related to the axis of time. They are to compare the value of asbestos fibre concentration.

Figure 3 shows the pollution value of the examined buildings in two categories of asbestos fibre concentration, in the range < 300–750 f/m^3^ and above 1500 f/m^3^.

## Data analyses and discussion

**Figure 4:**
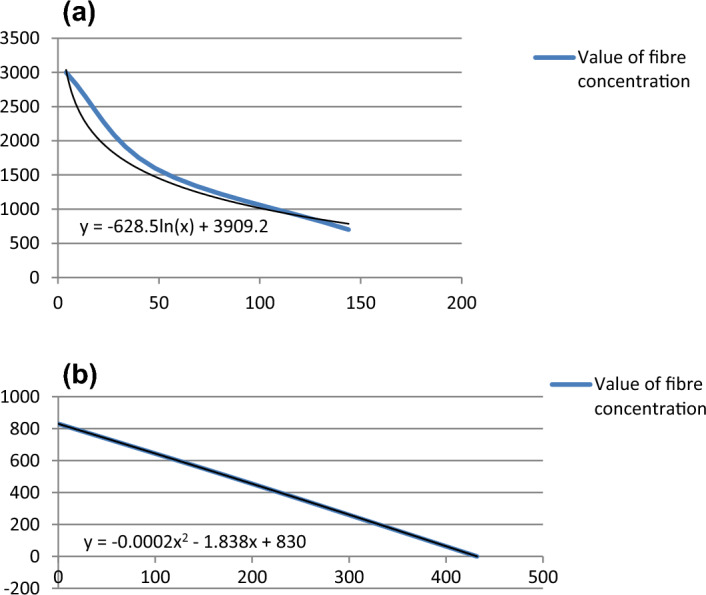
(**a**) Trend of changes in Building no. 1 over time. The X-axis is the elapsed time [in weeks], and the Y-axis is the concentration of respirable asbestos fibres [f/m^3^]. (**b**) Trend of changes in Building no. 2 over time. The X-axis is the elapsed time [in weeks], and the Y-axis is the concentration of respirable asbestos fibres [f/m^3^].

**Figure 5:**
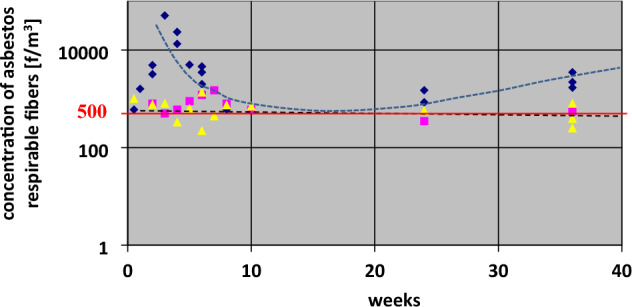
Changes in concentration of internal air pollution in “LIPSK” buildings during use and asbestos removal. Comparison of three buildings over a 40-week period. Filled diamond Asbestos dismantling. The increase in indoor air pollution after the work (after the 10th week) was caused by the drying of the walls. Filled square Normal room operation and Filled triangle Normal room operation (with standard operational ACM damage. After the tenth week from the start of the research, measures limiting the destruction of products and dust emission were introduced in the use of the buildings).

**Figure 6:**
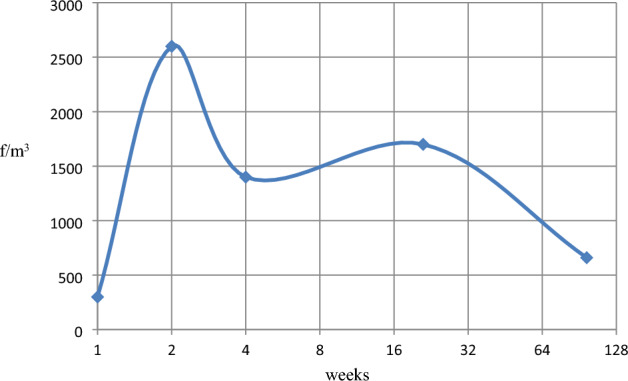
Estimated balance of asbestos-borne changes in rooms outside the work zones of “LIPSK” and “BERLIN” buildings subject to the asbestos removal process, taking into account routine execution errors.

Due to the location and time of "area sampling", the author’s results are lower than the results of "individual samples" and "area samples" reported in the cited literature (Tables 1 and 2). This is the result of the distance between the dust source and the air sampling sites. In addition, the passage of time between the destruction of the ACM and the tests performed by the author is important. This is visible in the graph, Figs. 2,4,5 and 6.

Comparing similar categories of buildings, described as in use, regularly serviced, in good condition, and without significant ACM damage, the differences between the presented results and the literature's results (type measurement 1 in Table 1) can be ignored. They are relatively small. It can be expected that this is due to natural processes that result from the long-term use of products in the correct way (air exchange, settling, etc)^37,38^. In the literature’s studies conducted with the use of the TEM technique in used buildings, asbestos fibre concentration values ranged between 50 and 120 f/m^3^^23,24^. They corresponded to the results of the data obtained through the OM technique. Here, values of about < 300 f/m^3^ were reached. This is a value below the limit of quantification for the OM method used. These values are insignificant compared to the values discovered after asbestos removal and are named here as a separate “5th asbestos risk category” (see Table 1). The levels of contamination after accidental ACM damage and careless or erroneous asbestos removal during renovation works were found far beyond the work zones, and also during the use of the buildings. These are values of about several thousand f/m^3^ with a range ≈ of 2000–6000 f/m^3^. That exposure applies not to contractors but to occupants, users and support staff. Data from research on Korean preschool buildings (Tables 1 and 2)^22^, although similar to the obtained values, do not ensure that we are considering the same, counted (asbestos) fibres. Identification was not used in the Korean study, contrary to the presented methodology.

The TM measurement made by Lee and Van Orden et al.^23^ should be considered significant and more comparable to the data discussed here. They state that among the several thousand samples tested from hundreds of US buildings, no values above 4000 f/m^3^ were detected. Therefore, a conclusion was drawn that the permissible limits were not exceeded and that the users were not at risk.

The results clearly prove that the time elapsed between activities likely to violate ACMs and the air sampling affects the recorded values of building pollution. It results from changes in the value of dust concentrations that occur over time^37,38^.

Improperly conducted works are accompanied by the uncontrolled emission of asbestos dust at the place of its generation. Mainly due to the dispersion of dust in the environment (in fresh air), fibre concentration drops at the place of its generation. On the other hand, asbestos-contaminated space grows behind the workplace. That contamination is not compulsorily controlled. As a result, there is an increased risk of building occupants being exposed to asbestos and they are not aware of the health risks involved.

Some rooms in buildings nos. 3a and 4a were continuously used for more than 40 years. The ACMs in poor condition, with visible damage to ACMs caused by thermal and humidity changes of external walls can cause varying effects on indoor air. The damages in building no. 1 and 3a, and 3b are similar, but the results of pollution measurements are different. However, despite similar damage, most of the 60 measurements in buildings 3a and 4a showed no asbestos in the air. The average value was < 300 f/m^3^. Maximum contamination 560 f/m^3^. The presented studies indicate that local operational damage to the ACMs, in normally used buildings with asbestos, does not cause an increase in the concentration of asbestos dust in the air above 500 f/m^3^. Larger contamination than 1000 f/m^3^ may cause large-scale damage to these products, related to asbestos removal, renovation and adaptation of rooms. In addition, activity in such rooms is necessary to keep asbestos dust suspended in the air.

The average test results in rooms with the poor technical condition of ACMs and work errors were characterised by a large dispersion of fibre concentration at the Std. Dev. level σ ≈ 50% for the average value of the obtained results as an effect of different sources of dust emission.

Changes in the concentration of dust over time are shown in Fig. 4a and b.

The blue line presents the concentration of asbestos dust, black corresponds to the trend of changes in these values. With the existing theoretical model of contamination variations (Fig. 4a), resulting from the course of changes according to the time scale, the concentration of asbestos dust in building No. 1 will reach the value of < 300 f/m^3^ in approximately three years from the termination of improperly conducted works. It should be emphasised that the conducted works only slightly and accidentally damaged the surface structure of the ACMs in comparison to asbestos removal work. The maximum values of air pollution during wrong renovation (in Building No. 1) were at least ten times lower than during complete asbestos removal operations in similar buildings. This is determined by the scale of the destruction^35^. During the works (in time “0” on Fig. 4a), indoor air contamination could be expected in the range of 7000–10,000 f/m^3^ behind the work zone. This accounts for approximately 1/10 of the PEL. Compared to historical Danish data, the estimated values for such renovation would exceed three times the lower occupational exposure range recorded in these years^45^.

Comparing the two diagrams (Fig. 4a and b), which present trends of changes in asbestos dust concentration over time (black curves), we observe that the high rate of decrease in the initial period for Building No. 1 corresponds to the low change in the air in Building No. 2. After two years, both models of changes in air pollution (Fig. 4a and b) became similar to each other.

In Fig. 4b after years, contamination is at the level of < 300 f/m^3^ (in the OM studies) or < 170 f/m^3^ (in the SEM–EDS studies). This corresponds to Buildings Nos 3, 4 and 5, containing ACM products in very good technical condition, which have not been disturbed in the past.

In the case of the asbestos disassembly process in buildings type "LIPSK" and the levels of dust leakage from work zones recorded here, one can expect an increase in fibre concentration in the zones not covered by the works, on average up to 3000 f/m^3^ or more. This value is also confirmed by Fig. 5^37^.

Using Fig. 2 and measurements during renovations^37,38^, Fig. 6 was developed. It shows the condition of 14 typical average buildings ("LIPSK") during and after asbestos removal in the rooms behind the work zones. In the chart, the work was completed after the second week and the average building was cleaned by the fourth week of work.

The effects of the asbestos removal process after the finished works can also be seen in Fig. 2. The pollution values of the “LIPSK” buildings after asbestos removal are marked with a red circle.

The analysed example of "MOA" building No. 11 shows that the removal of building partitions (roof and walls) immediately reduces the exposure of workers to asbestos by half. “Interior” contamination obtains background value within days to a week. This can happen almost immediately after removing the barriers between indoor and outdoor air and is the obvious effect of transporting and diluting pollutants in the "open space". This is reprehensible but possible.

## Assuming

When discussing the risks associated with the removal of asbestos from buildings, it is worth mentioning the discourse on the increase in the incidence of diseases as a result of occupational and para-occupational exposure caused by the removal of asbestos-cement products. The predicted increase in diseases^46^ has been estimated at several thousand cases in the country by 2030. In opposition to this thesis^47^ such a scenario was considered unbelievable, because values measured in the outside air were in the range of 500–1000.

Disregarding the methodology of disease forecasting, which the author does not undertake to evaluate, the author’s results show, about 10 times higher values of asbestos fibres concentration for indoor air after the incorrect works.

Moreover, using electron microscopy techniques one can expect to additionally double this value (due to the higher resolution and magnification of the electron microscopic image)^44^. This state of affairs is due to the lack of appropriate provisions in the national legislation, which tolerates low-quality asbestos dismantling works. Furthermore, no appropriate limit of asbestos air contamination inside the buildings (especially after the removal of ACM products) has been considered in that legislation. However, a permissible value in using rooms with ACM products and installations is PEL = 0.1 f/ccm.

And yet this value is (and should be only) reserved for the process of dismantling asbestos in airtight work zones with specialist dust protection of the building and contractors. For comparison^48^, German regulations set such a limit at a level 200 times lower.

The threat of dismantling and disturbance of ACMs poses a risk of increasing the concentration of asbestos dust in the air. Based on a databases of historical workers’ exposure to asbestos dust^17,36,44^, the highest airborne fibre concentrations were registered during construction services' active handling of asbestos products. The lowest personal exposure concentrations were observed in processes that do not require the manipulation of products.

This fact leads to reflection on whether the absolute removal of asbestos from buildings is the right trend in construction policy.

## Conclusions

1. The concentration of airborne asbestos in buildings does not have a fixed value. It changes over time due to many factors. For this reason, single, detailed measurements do not constitute a reliable risk assessment for building users.
2. Contamination from defective asbestos work by specialists is comparable to DIY and can involve the periodic, sometimes long-term, contamination of a building.
3. Renovation of small ACM surfaces, (for example painting the walls whilst causing accidental mechanical disturbance of friable asbestos products) yields a large variation of respirable asbestos fibre concentrations in rooms. The values of asbestos concentration depend on the distance of the sample from the emission source and are not repeatable.
4. These values significantly exceed the average concentration of asbestos fibres in buildings containing similar ACMs in normal use and after the correct removal or preservation of ACMs. At the same time, the average value of airborne asbestos in this case is many times lower than that generated during the dismantling of products in work areas.
5. All improper work carried out outdoors involves a "short-term" increase in pollution in the ambient outdoor air. The elevated dust concentration in outdoor air is short-lived and decreases rapidly with increasing distance from the dust source.
6. The speed of asbestos fibre concentration changes in the building depends on the degree of air exchange and the passage of time between the release of fibres from the ACMs and air sampling for the tests.
7. Some factors such as the leakage of fibres from sealed work areas, the free flow of dust outside the work area, dust dispersion in the environment, airing the rooms immediately before testing, and the passage of time between removal and air tests can affect the final opinion of the quality of asbestos removal work.

### Supplementary Information

Supplementary Information 1.

Supplementary Information 2.

Supplementary Information 3.

Supplementary Information 4.

## Data Availability

All data generated or analyzed during this study are included in this published article [and its supplementary information files]. The raw and unanalyzed dataset components used in the article are not publicly available [due to the number and volume of information], but are available from the author upon reasonable request.
