# Supplementary material for: Comparison of the effects of use, protection, improper renovation and removal of asbestos products on the example of typical old office buildings in Poland
Source: Sci Rep. 2023 Aug 21;13:13577. doi: 10.1038/s41598-023-37257-z (PMC10442424; doi:10.1038/s41598-023-37257-z)
Supplement: Supplementary file 1 — Supplementary Information 1. [file 41598_2023_37257_MOESM1_ESM.docx]

Damage to “SOKALIT” boards **Appendix A1**


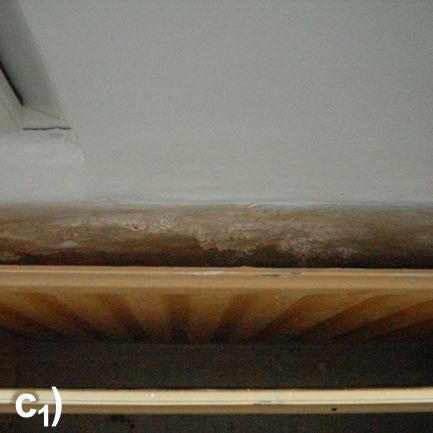

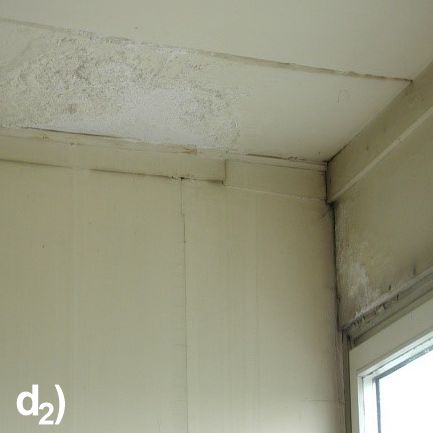

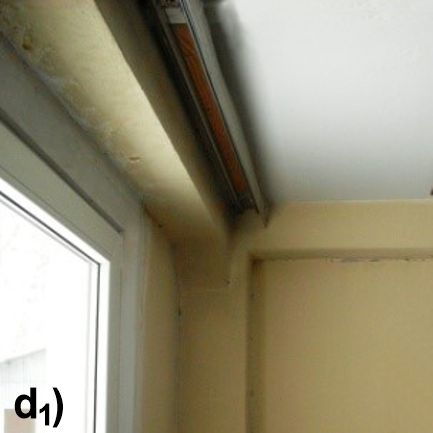

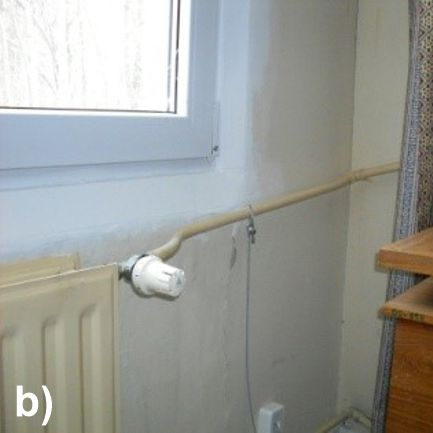

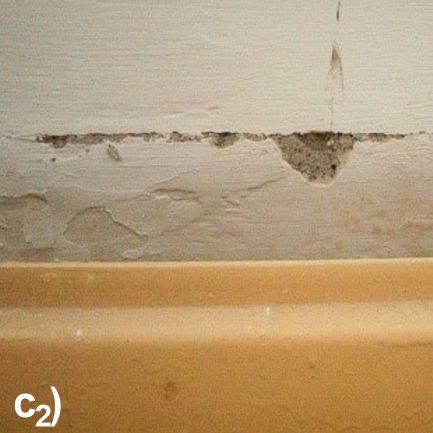

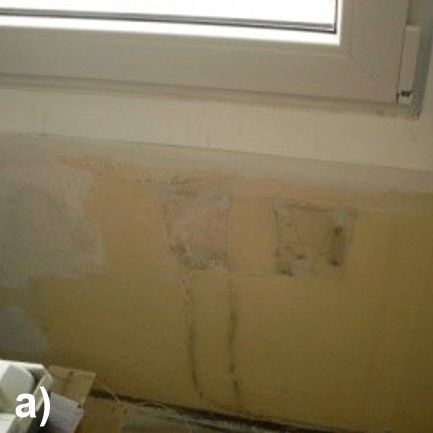


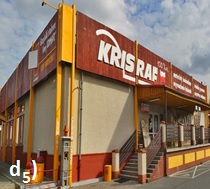

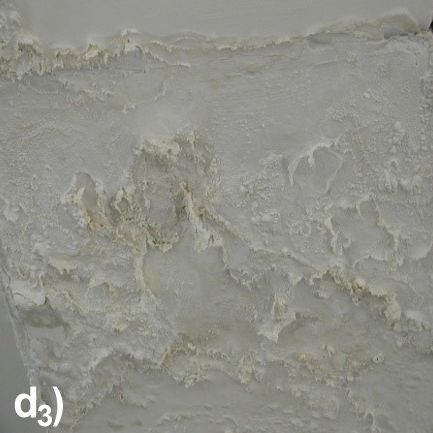

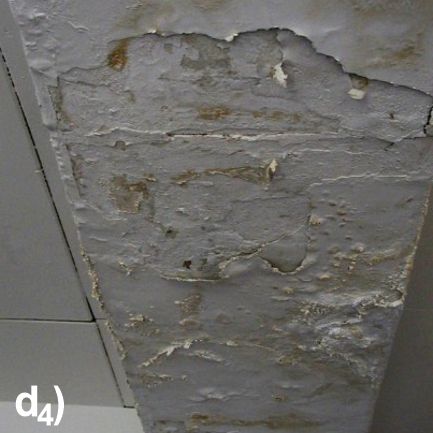


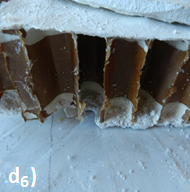

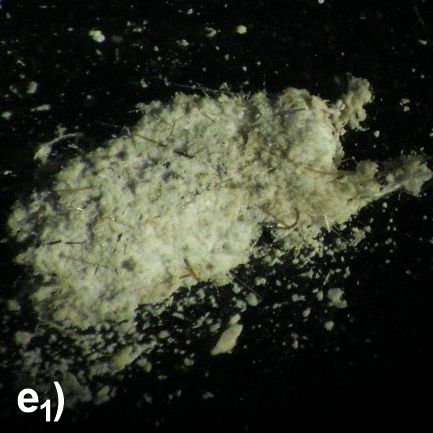

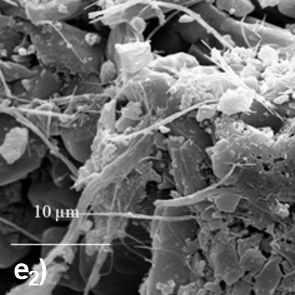


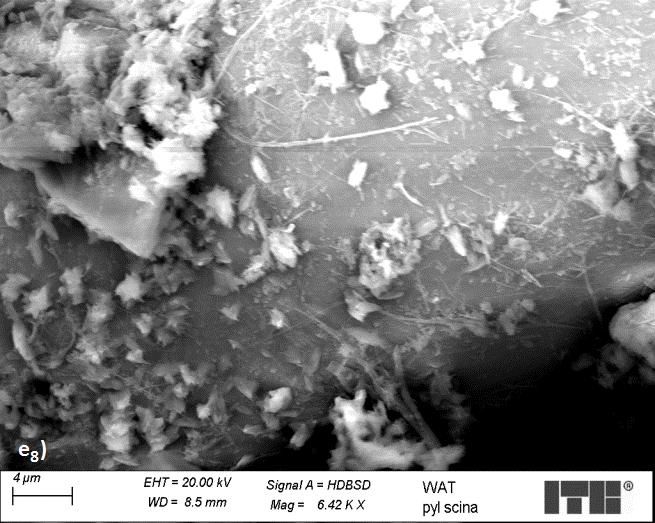

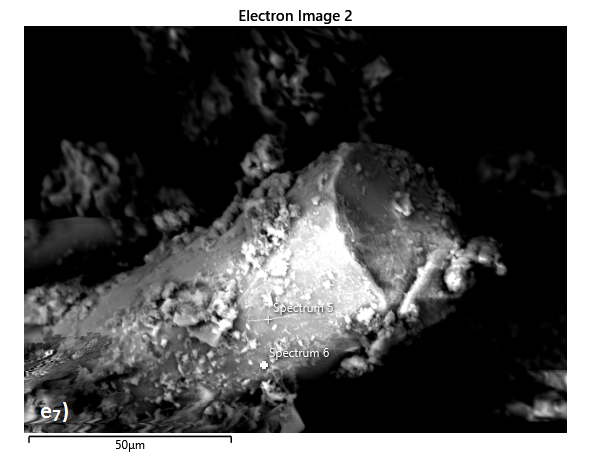

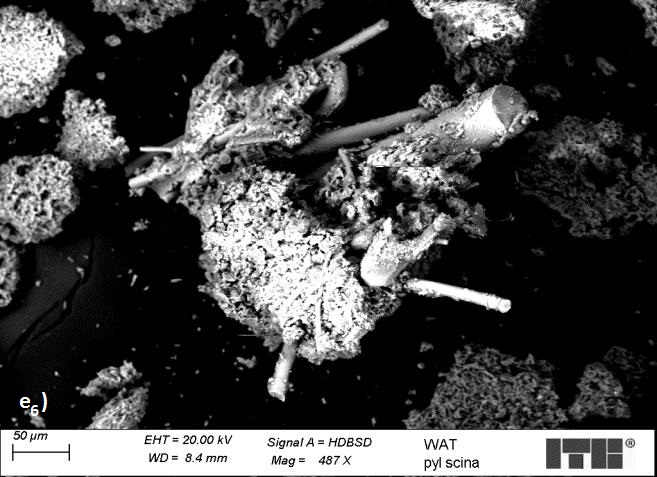


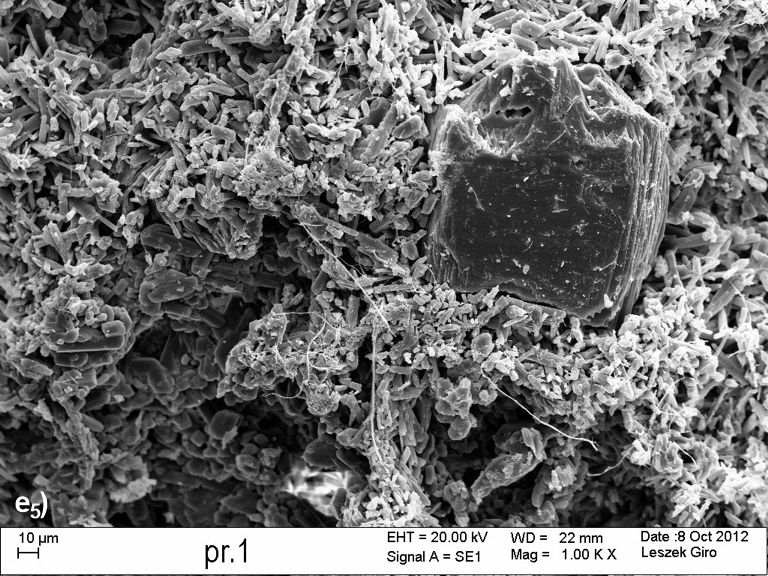

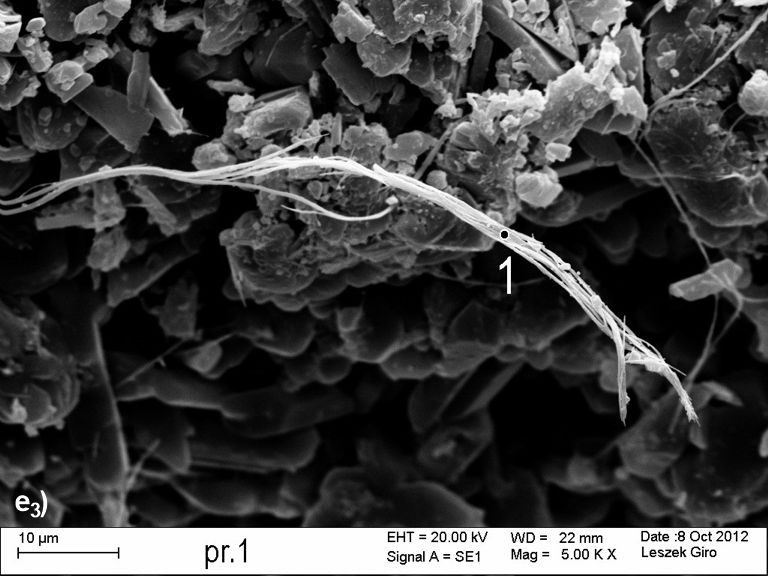

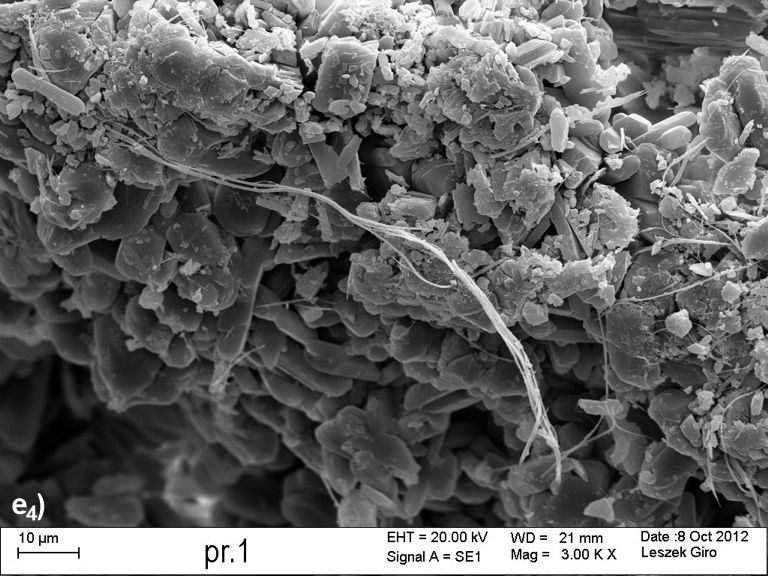


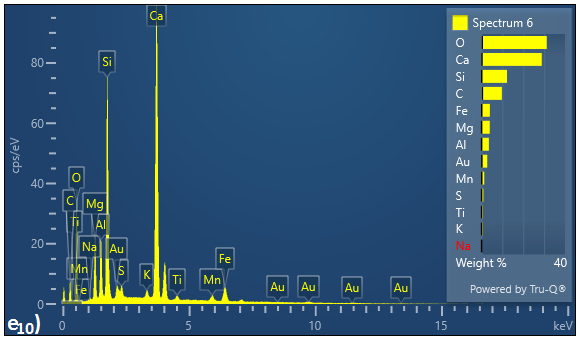

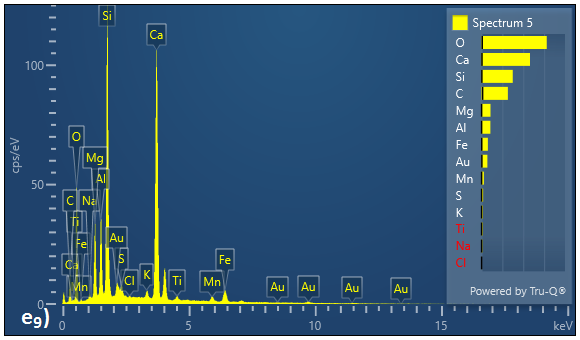


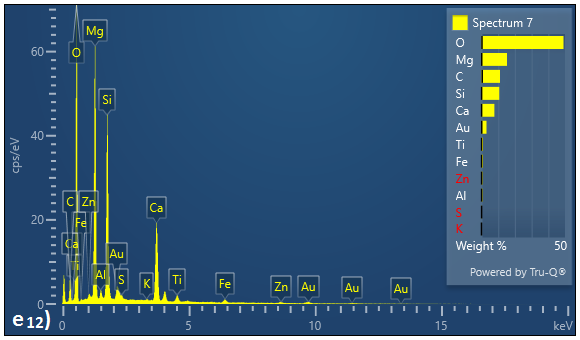

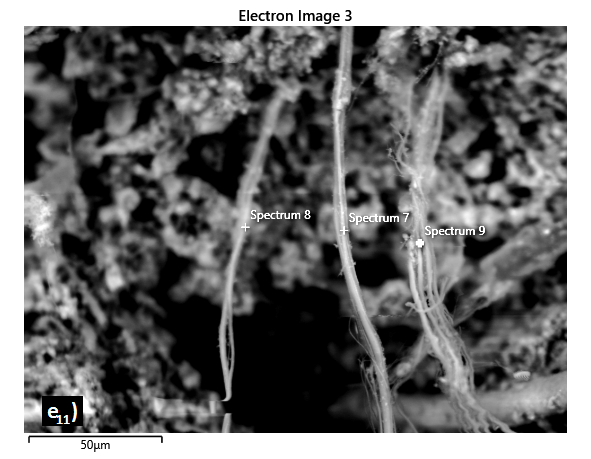


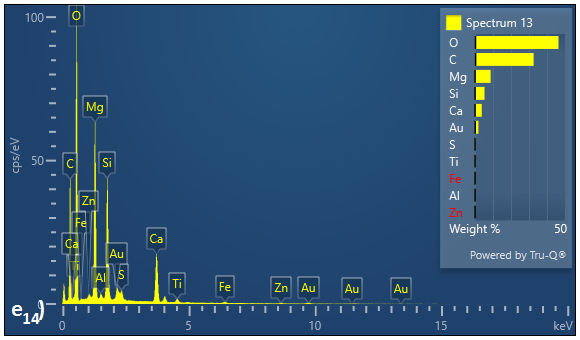

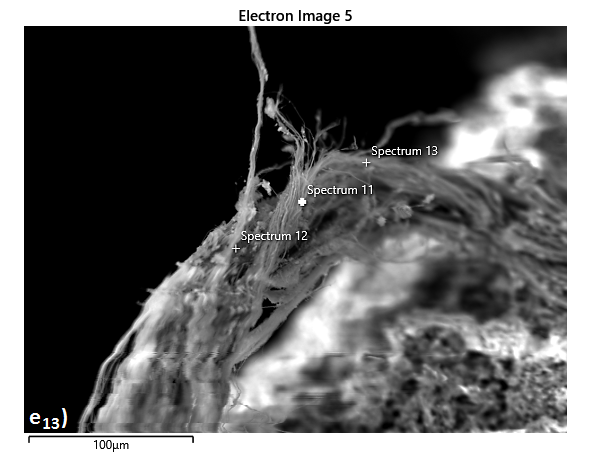


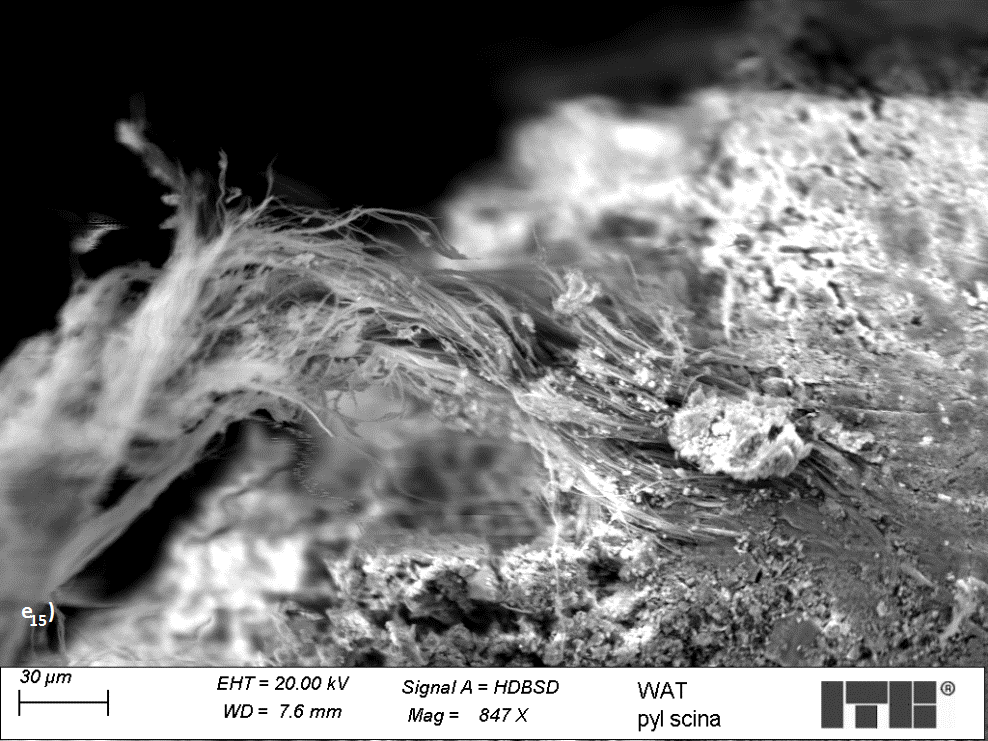


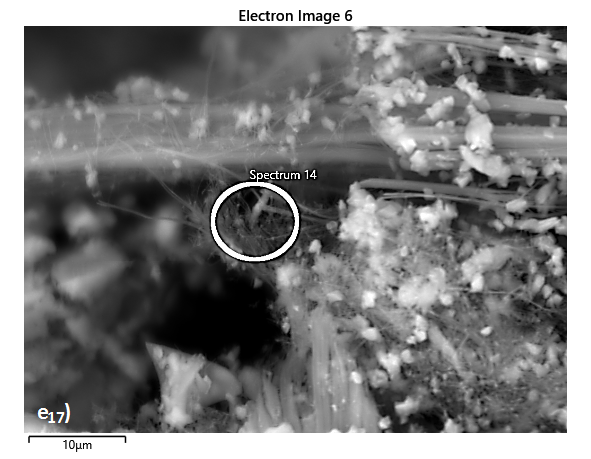

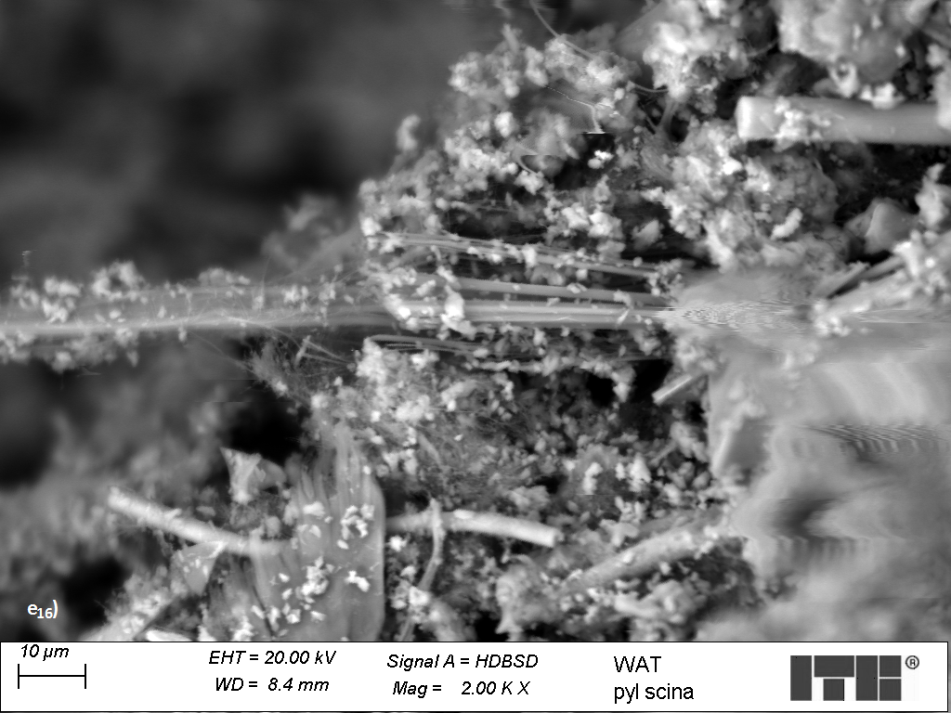


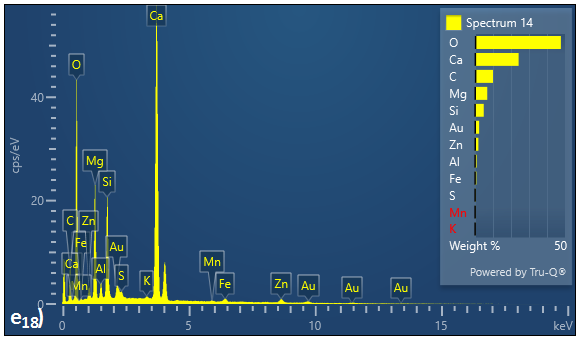


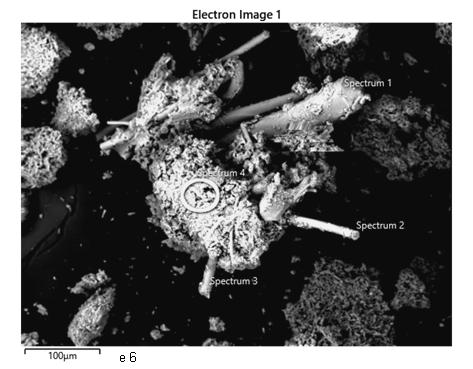


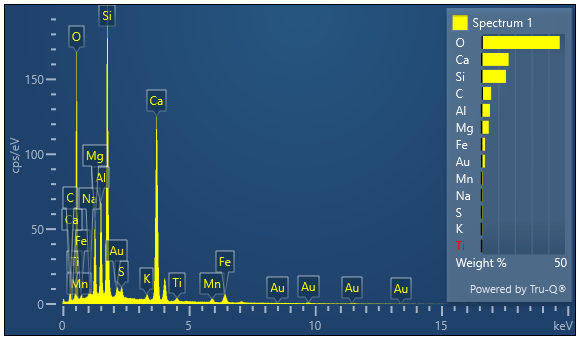

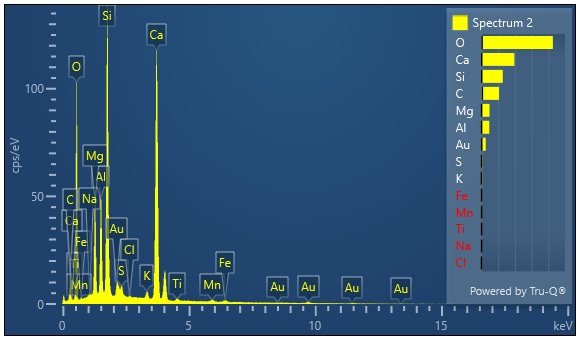


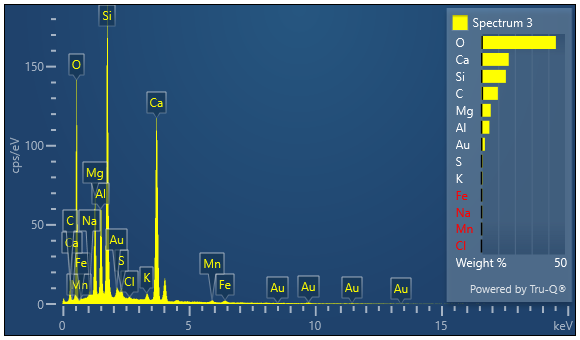

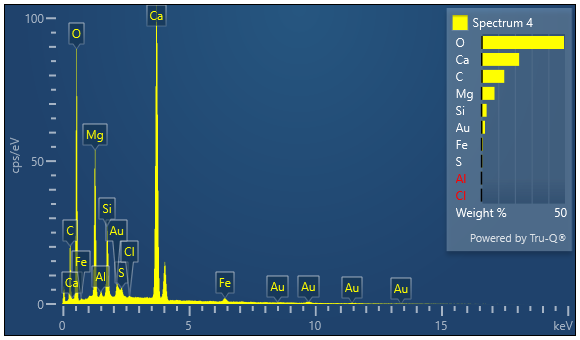


Damage to the ACM in the "BERLIN" building. The type of damage to the ACM was caused by the freezing of the walls and condensation of moisture on the inner surfaces of the walls (d_2_, d_3_ and d_4_). That typical damage was visible in rooms in building no.1. Cases visible in photos c_2_ and d_1_ were caused by natural mechanical damage and vibration of the building structure.

a) b) Mechanical damage before painting, after replacing windows.

c_1_) Destruction of the friable ACM, "SOKALIT" panels behind the radiators (caused by changes in moisture ).Enlarged fragment of damage to the "SOKALIT" panels around the frames above the radiators at the panel joints.

c_2_) Gaps on the SOKALIT panel joints behind the radiator. Damage caused vibration of a non-rigid building structure and, cracks in wall elements of different stiffness.

d_1_) Damage to the walls caused by ageing and structure vibrations: Destruction of the "SOKALIT" boards in the vicinity of the lintels above the windows; Damage to the "SOKALIT" panels at the point of contact between the partition wall and the building structure.

d_2_), d_3_) and d_4_) Fragments of ceiling surfaces and wall corners made of "SOKALIT" degraded by water penetration (steam condensation, wall freezing, sewage pipe leakage), typical damage in buildings type “BERLIN” or “LIPSK”.

d_5_) Shop pavilion Type C III manufactured by the GDR, made at the same time and with the same materials as the "BERLIN" building.

d_6_) A particle of a curtain wall, also used in partition walls in buildings Type BERLIN and Type C III

e_1_) Product of corrosion and destructive water damage to the "SOKALIT" board. Crushing fragments of "SOKALIT" boards collected from the floor under radiators. e_2_) The dust that collects on the floor at the site of damage and its image seen in SEM, magnification 1000 x.

e _2_) – e_17_) Analysis of the composition of dust collected on the floor in rooms, near the walls. Dust samples are presented in Appendix A1, e_1_) and A2, b). The dust grains are dominated by elements of gypsum-lime mortar with fragments of glass fibres (e_6,_ and e_7_). High magnification of the grain in photo e_7_ (visible in photo e_8_) revealed thin asbestos fibres on the surface of the fibreglass. In the remaining photographs (e_2_, e_3_, e_4_, e_5_ and e _11_, e _13_, e _15_, e _16_ and e _17_) the EDS analysis showed the presence of chrysotile asbestos.
