# Supplementary material for: Comparison of the effects of use, protection, improper renovation and removal of asbestos products on the example of typical old office buildings in Poland
Source: Sci Rep. 2023 Aug 21;13:13577. doi: 10.1038/s41598-023-37257-z (PMC10442424; doi:10.1038/s41598-023-37257-z)
Supplement: Supplementary file 2 — Supplementary Information 2. [file 41598_2023_37257_MOESM2_ESM.docx]

**Appendix A2**

Comparison of similar damages of ACM-s products in "BERLIN" types buildings no. 1 and nos. 3a and 4a

**Building „BERLIN” no. 1**


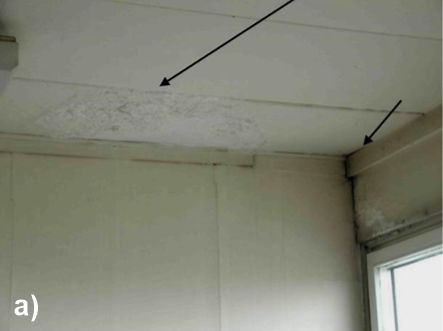

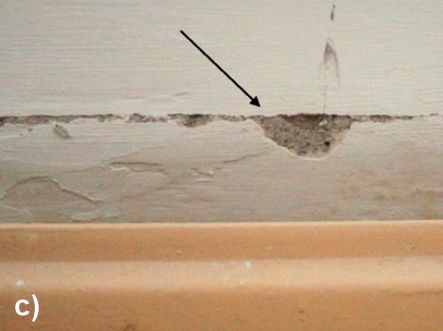

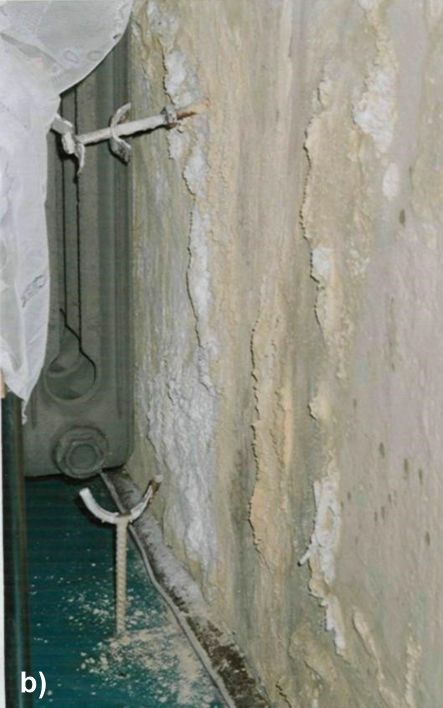

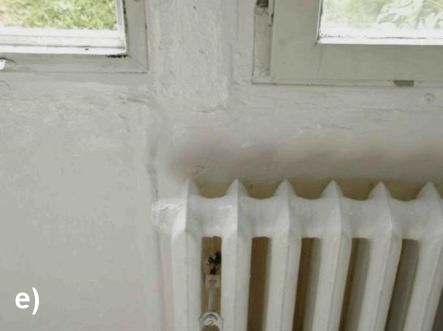

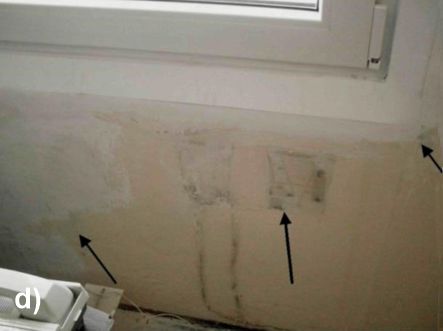


Photos a) – e) present the condition of asbestos-containing products ("SOKALIT") in building No 1 at the time when the renovation was stopped and the tests began. Airborne concentration in that room was about 3200 – 7000 f/m^3^

a) A leaky roof damaged the suspended ceiling with “SOKALIT” (big arrow). Biological destruction (fungal infestation) of the corner of the window strip and the gable wall by freezing the walls (Photo 1a, small arrow and 1b, photo below ).

b) Damage caused in an unused room on the inside of the outer wall of the building (the effect of freezing and condensation)

c) Paint cracks on the joints of “SOKALIT” boards (arrow) visible behind the edge of the radiator (caused by vibrations of the building structure and local temperature changes).

d) Damage and defects of the “SOKALIT” surface covered with plaster mortar.

e) Destruction of the plate “SOKALIT” surface, which develops under the paints under ad hoc repairs. Inappropriate paints were used that do not match the surface of the plate

**Buildings “BERLIN” nos. 3a and 4a**


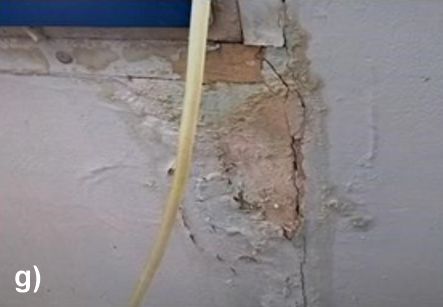


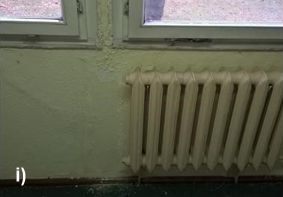

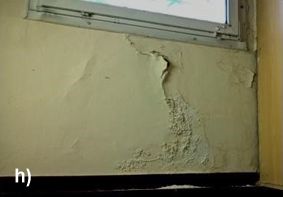

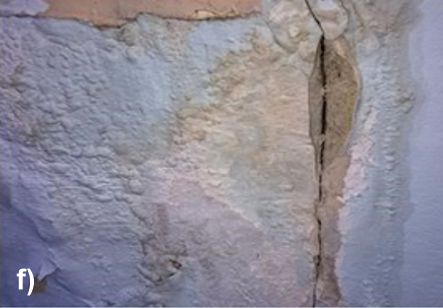


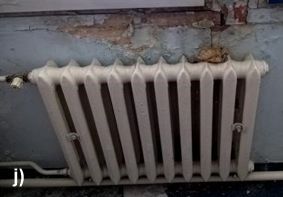


Photos: f) – j) present the condition of asbestos-containing products ("SOKALIT") in buildings nos. 3a and 4a during normal use as classrooms in the school. The average value of 60 measurements of the concentration of asbestos fibres in the air in both buildings was < 300 f/m^3^. The maximum value was 560 +/- 60 f/m^3.^
