# Supplementary material for: Comparison of the effects of use, protection, improper renovation and removal of asbestos products on the example of typical old office buildings in Poland
Source: Sci Rep. 2023 Aug 21;13:13577. doi: 10.1038/s41598-023-37257-z (PMC10442424; doi:10.1038/s41598-023-37257-z)
Supplement: Supplementary file 3 — Supplementary Information 3. [file 41598_2023_37257_MOESM3_ESM.docx]

**Appendix B1**

Demolition of the MOA building. The general appearance of the work zone.


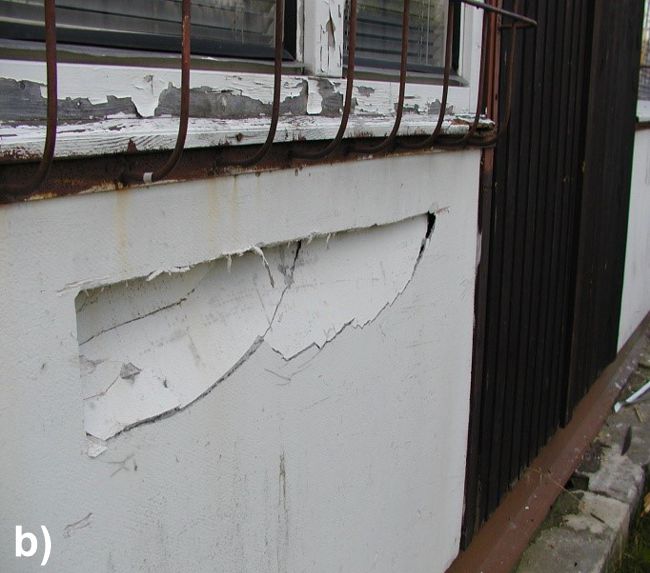


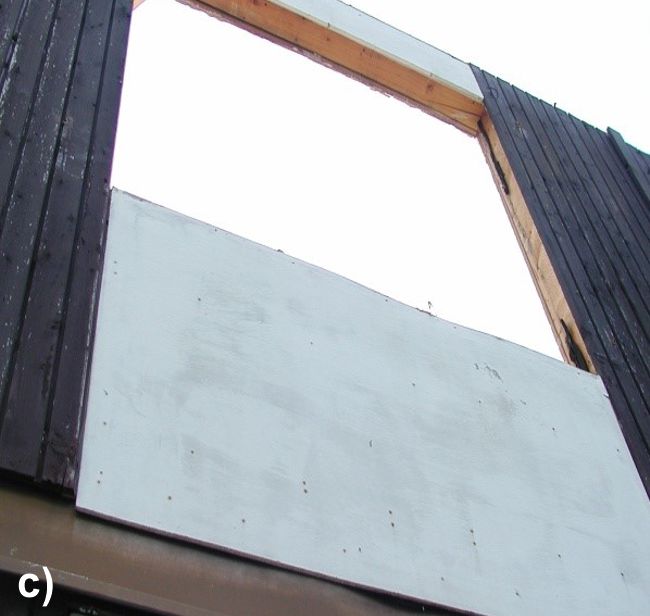

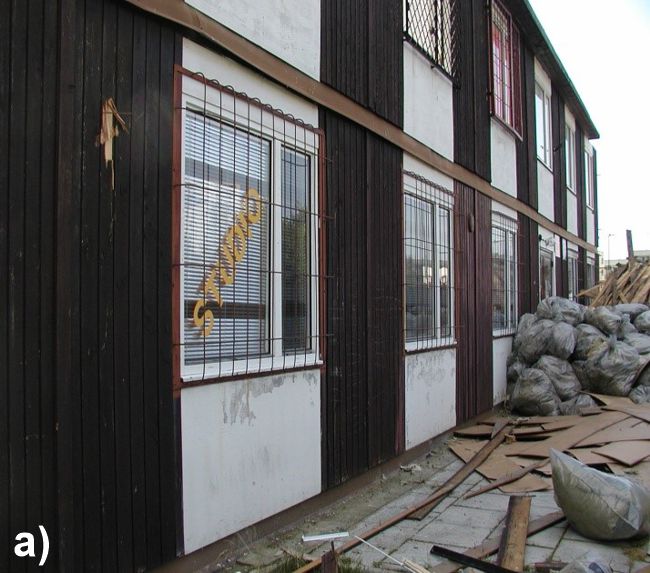


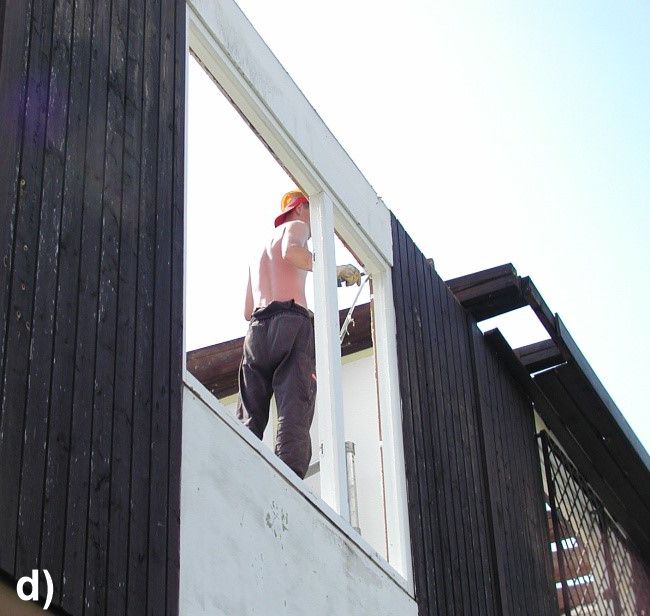


a) Lack of segregation of waste, mixing of asbestos with other waste, lack of sealing of waste.

b) Destruction of the ACM panels that make up the internal and external sheathing of the building's external and partition walls.

c) View of a section of the external wall on the 1st floor.

d) View after removing the roof. The14^th^ day of work.
