# Supplementary material for: Comparison of the effects of use, protection, improper renovation and removal of asbestos products on the example of typical old office buildings in Poland
Source: Sci Rep. 2023 Aug 21;13:13577. doi: 10.1038/s41598-023-37257-z (PMC10442424; doi:10.1038/s41598-023-37257-z)
Supplement: Supplementary file 4 — Supplementary Information 4. [file 41598_2023_37257_MOESM4_ESM.docx]

**Appendix B2**

MOA building during demolition, after removing the roof.


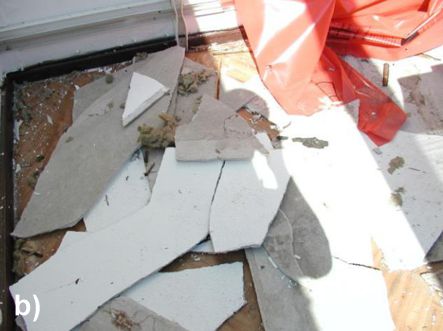


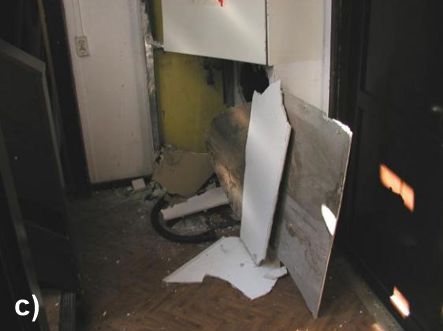

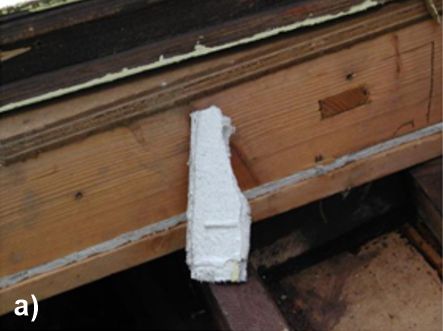


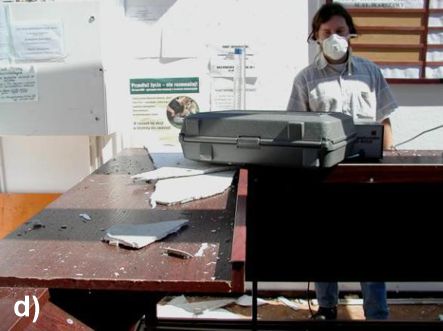


The photographs show the scale of irregularities that result in the environmental pollution described in the text.

The air samples were taken on the ground floor. The sun traces visible in the photographs (b, c, d) are evidence of the removal of the roof and ceiling together with the floor of the first floor. The interior of the building became open to the outside air.

1. Sandwich wall with crushed friable ACM (whiteboards) in the curtain wall of the demolished building.
2. and c) The fragments of ACM boards (similar to "SOKALIT") were scattered around the interior of the rooms immediately after the roof was demolished.

d) Air sampling by the ITB research team inside the building that is being disassembled.
